# Evaluation of the accuracy of cone-beam CT–based dose calculation for target volumes and organs at risk in left-sided breast cancer radiotherapy

**DOI:** 10.3389/fonc.2026.1768319

**Published:** 2026-03-03

**Authors:** Xiaoxiao Hou, Zhenglu Bai, Xiaoxiao Jin, Erxun Dai, Yaqi Jiang, Jun Ma, Jun Li

**Affiliations:** Radiotherapy Center, Northern Jiangsu People's Hospital, Yangzhou, Jiangsu, China

**Keywords:** breast cancer, cone-beam CT, dosimetric evaluation, organs at risk, radiotherapy

## Abstract

**Introduction:**

Accurate dose calculation is essential in breast cancer radiotherapy. This study aimed to evaluate the feasibility and accuracy of cone-beam CT (CBCT) images with HU–relative electron density (HU-RED) correction for dose calculation in left-sided breast cancer radiotherapy.

**Methods:**

Twenty postoperative patients receiving adjuvant radiotherapy at Subei People’s Hospital of Jiangsu Province (Yangzhou, China) from January 2022 to December 2024 were retrospectively enrolled. A patient-specific HU-RED calibration curve was generated using an improved density override method. Treatment plans created on planning CT (pCT) were transferred to CBCT for dose recalculation with identical optimization parameters. Dosimetric parameters of the planning target volume (PTV) and organs at risk (OARs) were compared. Two-dimensional gamma analysis was performed to assess dose consistency. Equivalence between CBCT- and pCT-based dose calculations was further evaluated using the two one-sided tests (TOST), Lin’s concordance correlation coefficient (CCC), intraclass correlation coefficient (ICC), and Bland–Altman analysis.

**Results:**

Differences in PTV dosimetric indices (D2, D50, D98, Dmean, HI, CI) between CBCT- and pCT-based plans were small, with mean deviations <1.3% and no statistically significant differences (*P* > 0.05). OAR parameters, including cardiac Dmean, V20, V30, V50 and lung Dmean, V20, V30, also showed minimal variation (<1.5%), with the largest deviation observed in cardiac V20 (2.1% in a single case). Gamma analysis revealed high agreement between both plans, with passing rates exceeding 90% for both the 3%/3 mm and 2%/2 mm criteria. Equivalence testing demonstrated statistical equivalence between CBCT- and pCT-based dose calculations for all PTV parameters and most OAR metrics. All 90% confidence intervals fell entirely within predefined equivalence margins (Δ), and all TOST *P*-values were <0.05. Lin’s CCC and ICC(A,1) exceeded 0.96 for all parameters, indicating excellent consistency, while Bland–Altman analyses showed minimal bias and narrow limits of agreement.

**Discussion:**

CBCT images corrected with HU-RED calibration achieved highly consistent dose calculation results compared with pCT in left-sided breast cancer radiotherapy. This method is feasible for clinical dose verification and may support future adaptive radiotherapy strategies, particularly with the integration of artificial intelligence techniques.

## Introduction

Breast cancer is the most common malignancy among women worldwide, accounting for nearly one-third of all female cancers, with a mortality rate of approximately 15% of diagnosed cases (1). Radiotherapy plays a critical role in comprehensive treatment, reducing local recurrence and improving survival outcomes (2). In radiotherapy planning, the fundamental objective is to ensure adequate target coverage while minimizing radiation exposure to normal tissues such as the heart and lungs, thereby improving therapeutic benefit and reducing long-term toxicity.

However, breast cancer radiotherapy is typically delivered over a prolonged treatment course. During this process, patient-related factors such as positioning, respiratory motion, and anatomical variations may lead to discrepancies between planned and delivered doses, potentially compromising treatment efficacy (3). The development of image-guided radiotherapy (IGRT) has provided novel approaches for improving treatment precision. Among IGRT modalities, cone-beam computed tomography (CBCT) has been widely applied due to its ability to acquire patient-specific anatomical information during treatment sessions. CBCT not only reflects the real-time status of targets and organs at risk (OARs) but also provides essential data for adaptive radiotherapy (4). Nonetheless, inherent limitations such as scatter artifacts, image noise, restricted field of view, and unstable Hounsfield unit (HU) values result in compromised image quality compared with conventional CT, raising concerns regarding the accuracy of CBCT-based dose calculation (5). Therefore, this study included 20 patients with left-sided breast cancer and compared CBCT-based and pCT-based plans in terms of dose-volume histograms (DVHs), isodose distributions, and gamma passing rates, aiming to assess the feasibility and accuracy of CBCT in dose calculation for breast cancer radiotherapy.

## Materials and methods

### Study population

Twenty postoperative patients receiving adjuvant radiotherapy at Subei People’s Hospital of Jiangsu Province (Yangzhou, China) from January 2022 to December 2024 were retrospectively enrolled (mean age: 43 ± 4.3 years). All patients had undergone breast-conserving surgery or modified radical mastectomy, with histopathological confirmation of stage I–III disease. Inclusion criteria: (i) postoperative left-sided breast cancer receiving conventional fractionated radiotherapy; (ii) availability of complete pCT, pre-treatment CBCT, and planning data; (iii) completion of full treatment course. Exclusion criteria: (i) poor image quality or severe artifacts; (ii) prior thoracic irradiation; (iii) severe cardiopulmonary comorbidities affecting dosimetric assessment. Ethical approval was obtained from the institutional review board.

### Radiotherapy regimen

All patients were immobilized in the supine position with both arms raised using vacuum cushions. Simulation CT was performed at 120 kV with 3 mm slice thickness. The prescription dose was 50 Gy in 25 fractions over 5 weeks. For selected patients, regional nodal irradiation (axillary/supraclavicular) was delivered with the same prescription. Radiotherapy techniques included intensity-modulated radiotherapy (IMRT) and conformal radiotherapy (CRT), ensuring that at least 95% of the planning target volume (PTV) received the prescribed dose.

### Equipment

Varian TrueBeam linear accelerator (Varian Medical Systems, USA); GE large-bore CT scanner (GE Healthcare, USA); Varian simulation localizer (Varian Medical Systems, USA); TPS version 15.6 treatment planning system; PTW Mephysto Navigator (PTW Dosimetry, Freiburg, Germany).

### Image acquisition and planning

CBCT scans were obtained prior to the first treatment session using on-board imaging. For pCT,images were acquired with a large-bore CT scanner (120 kV, 80 mA, 25 ms, slice thickness 5 mm). CBCT acquisition parameters included: 100 kV, 15 mA, 150 mAs, 2 mm slice thickness, 360° rotation, 650 projections, and a 40×40 cm² field of view. A patient-specific HU-RED calibration curve was constructed for CBCT dose calculation, while pCT plans were calculated with the standard HU-RED curve. All CBCT and CT images were rigidly registered based on bony landmarks, followed by manual verification and minor adjustments as necessary. For supplementary analysis, regions of interest (ROIs) were selected in lung, soft tissue, and bone on both pCT and CBCT images after rigid registration. The ROIs were placed in relatively homogeneous areas and matched in anatomical location between the paired image sets. Mean HU values were extracted for each tissue ROI. For RED analysis, the corresponding mean HU values from CBCT were converted to RED using the patient-specific HU–RED calibration curve and compared with those derived from pCT. The ROI-based HU and RED analyses are presented in **Supplementary Figures 1** and **S2**.

Treatment plans (IMRT and conformal radiotherapy [CRT]) were initially designed on pCT images and subsequently transferred to CBCT for direct dose recalculation, with all optimization parameters unchanged.

### Dosimetric evaluation

Dosimetric parameters for PTV included D2, D50, D98, Dmean, conformity index (CI), and homogeneity index (HI). OAR evaluation covered cardiac Dmean, V20, V30, V50 and bilateral lung Dmean, V20, V30, V50. Dose verification was performed with gamma analysis under 3%/3 mm and 2%/2 mm criteria, using a 10% dose threshold.

### Statistical analysis

All contours were independently delineated by two senior radiation oncologists and reviewed for consistency. Statistical analyses were performed using SPSS 26.0. Paired t-tests or Wilcoxon signed-rank tests were applied as appropriate.

To determine equivalence between pCT- and CBCT-based dose calculations, the two one-sided tests (TOST) procedure was conducted. Equivalence margins (Δ) were predefined according to clinically acceptable dosimetric tolerances (PTV: ± 0.5 Gy; OARs: ± 2%). Statistical equivalence was established when the 90% confidence interval of the mean difference fell entirely within Δ and the TOST P-value was <0.05. Lin’s concordance correlation coefficient (CCC) and intraclass correlation coefficient ICC(A,1) were calculated to assess agreement. Bland–Altman plots were generated to evaluate systematic bias and limits of agreement. Statistical significance was set at *P* < 0.05.

## Results

### HU-RED curves

**Figure 1:**
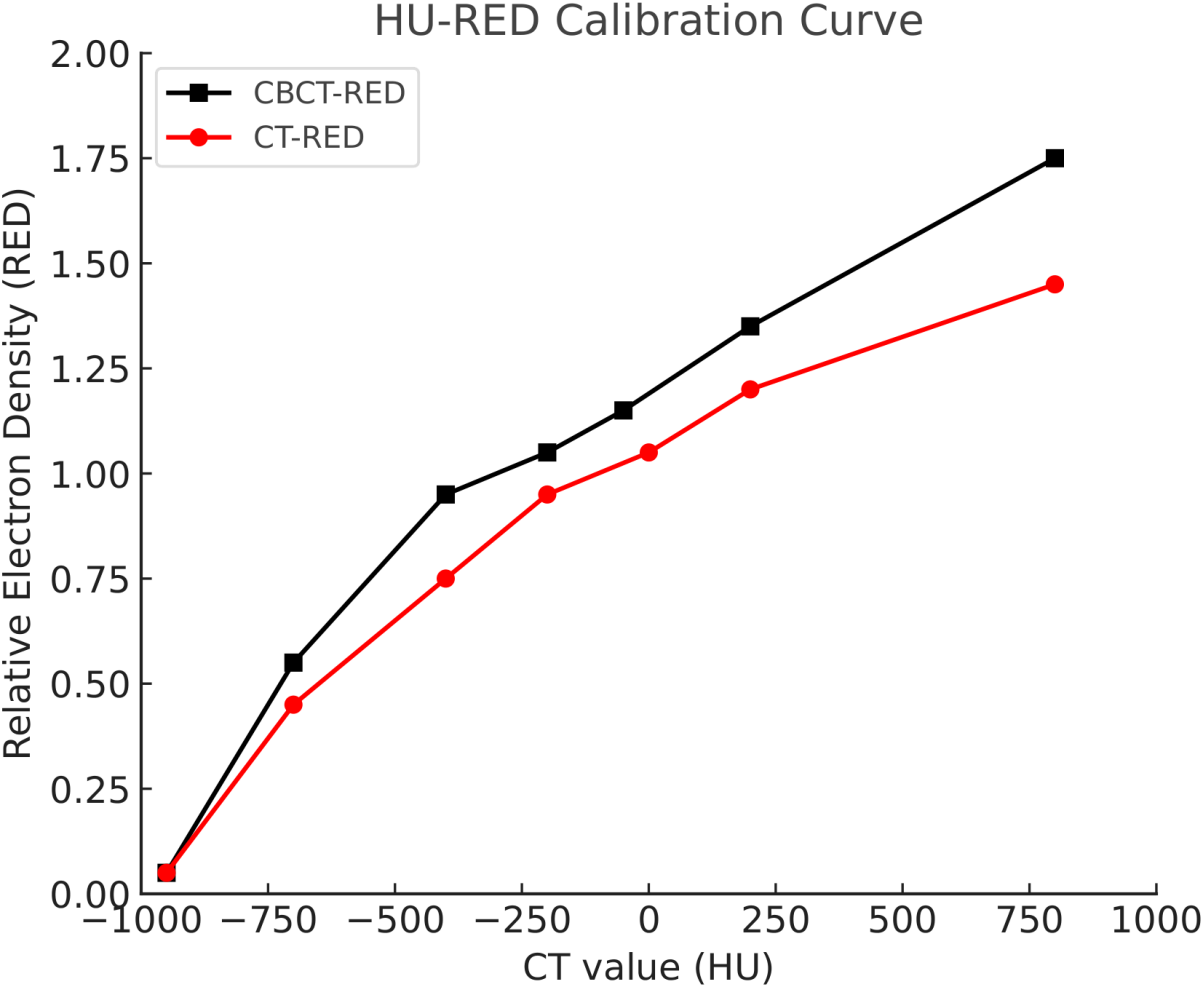
HU-RED curves.

CBCT- and pCT-derived HU-RED curves demonstrated similar trends, suggesting general consistency in tissue density quantification. However, greater HU discrepancies were observed in high-density tissues (e.g., bone) compared with low-density tissues (e.g., soft tissue and air) (**Figure 1**). In addition, ROI-based comparisons showed that the mean HU differences between pCT andCBCT for representative tissues (lung, soft tissue, and bone) were small, and the corresponding ROI-based RED differences derived from the HU–RED calibration were also limited (**Supplementary Figures 1**-**S2**).

### PTV and OAR dosimetric comparison

In IMRT, CBCT plans yielded slightly higher D2, D50, and Dmean, with marginally lower D98 compared with pCT; none of the differences were statistically significant (*P* > 0.05). In CRT, CBCT-based D2, D50, D98, and Dmean were slightly lower than those from pCT, also without statistical significance. CI and HI values showed minimal variation between the two modalities under both techniques (**Tables 1**, **2**).

**Table 1:** Statistical results of PTV dosimetric parameters in IMRT (mean ± SD).

PTV	D2/cGy	D50/cGy	D98/cGy	Dmean/cGy	HI	CI
pCT	5220 ± 110	5010 ± 70	4798 ± 95	5018 ± 64	0.085 ± 0.014	0.930 ± 0.020
CBCT	5230 ± 115	5006 ± 68	4791 ± 92	5022 ± 66	0.086 ± 0.015	0.920 ± 0.030
*t*	−0.281	0.183	0.237	−0.195	−0.218	1.24
*P*	0.78	0.856	0.814	0.847	0.829	0.222

**Table 2:** Statistical results of PTV dosimetric parameters in CRT (mean ± SD).

PTV	D2/cGy	D50/cGy	D98/cGy	Dmean/cGy	HI	CI
pCT	5280 ± 135	5018 ± 85	4755 ± 110	5025 ± 78	0.105 ± 0.018	0.900 ± 0.030
CBCT	5292 ± 140	5012 ± 82	4747 ± 112	5030 ± 80	0.106 ± 0.019	0.895 ± 0.032
*t*	0.38	−0.300	−0.520	0.42	0.22	−0.560
*P*	0.708	0.768	0.608	0.68	0.828	0.583

In both IMRT and CRT, CBCT and pCT plans exhibited non-significant differences in cardiac Dmean, V20, V30, V50 and bilateral lung dosimetric parameters. Differences were small in magnitude, with CBCT values occasionally slightly higher or lower than pCT values (**Tables 3**, **4**).

**Table 3:** Statistical results of OAR dosimetric parameters in IMRT (mean ± SD).

OAR	Indicator	pCT	CBCT	*t*	*P*
Heart	Dmean/cGy	420 ± 110	430 ± 115	0.524	0.606
	V50/%	0.6 ± 0.5	0.7 ± 0.6	0.746	0.465
	V30/%	2.6 ± 1.5	2.8 ± 1.6	0.654	0.52
	V20/%	5.1 ± 2.6	5.3 ± 2.7	0.388	0.703
Lung	Dmean/cGy	900 ± 220	912 ± 230	0.642	0.528
	V50/%	0.8 ± 0.6	0.9 ± 0.7	0.671	0.51
	V30/%	3.2 ± 1.6	3.4 ± 1.7	0.603	0.553
	V20/%	7.4 ± 3.1	7.7 ± 3.2	0.582	0.567

**Table 4:** Statistical results of OAR dosimetric parameters in CRT (mean ± SD).

OAR	Indicator	pCT	CBCT	*t*	*P*
Heart	Dmean/cGy	520 ± 130	528 ± 135	0.472	0.642
	V50/%	1.0 ± 0.7	1.1 ± 0.8	0.566	0.578
	V30/%	3.6 ± 1.8	3.7 ± 1.9	0.305	0.763
	V20/%	6.8 ± 2.9	7.1 ± 3.0	0.559	0.582
Lung	Dmean/cGy	1100 ± 260	1115 ± 270	0.591	0.561
	V50/%	1.2 ± 0.8	1.3 ± 0.8	0.544	0.592
	V30/%	4.2 ± 1.9	4.4 ± 2.0	0.487	0.632
	V20/%	9.0 ± 3.7	9.3 ± 3.8	0.469	0.645

### Difference in absolute values of dosimetric parameters between the two planned doses

In this study, the absolute differences in dosimetric parameters between CBCT-based and CT-based plans were generally minimal. For the planning target volume, the mean deviations in PTV D2, D50, D98, and Dmean remained within 1.5% across all patients, with the largest discrepancy observed in a single case for D2 (1.53%). The overall dose–volume distributions of the PTV on both image sets were highly comparable, and no clinically meaningful hotspots or cold spots were identified.

For organs at risk (OARs), the differences between the two planning methods were similarly small. Mean deviations were consistently below 1.75%, and the greatest variation occurred in the cardiac V20 parameter, which reached a maximum of 2.11% in one patient. The DVH curves of the heart and lungs demonstrated almost overlapping patterns between CBCT- and CT-based plans, further supporting the high level of dosimetric agreement.

**Figure 2:**
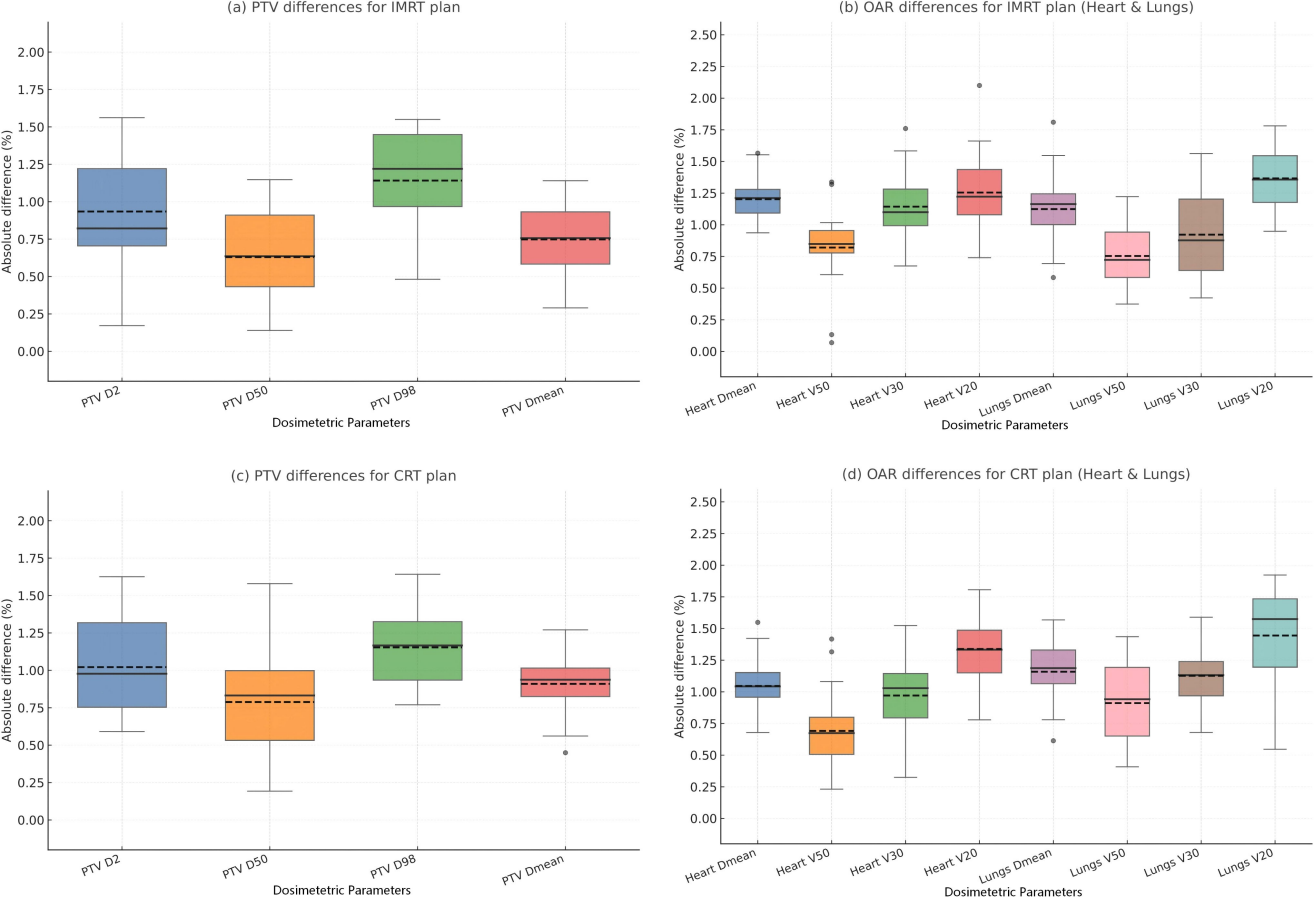
Absolute dosimetric parameters differences between two plans.

**Figure 3:**
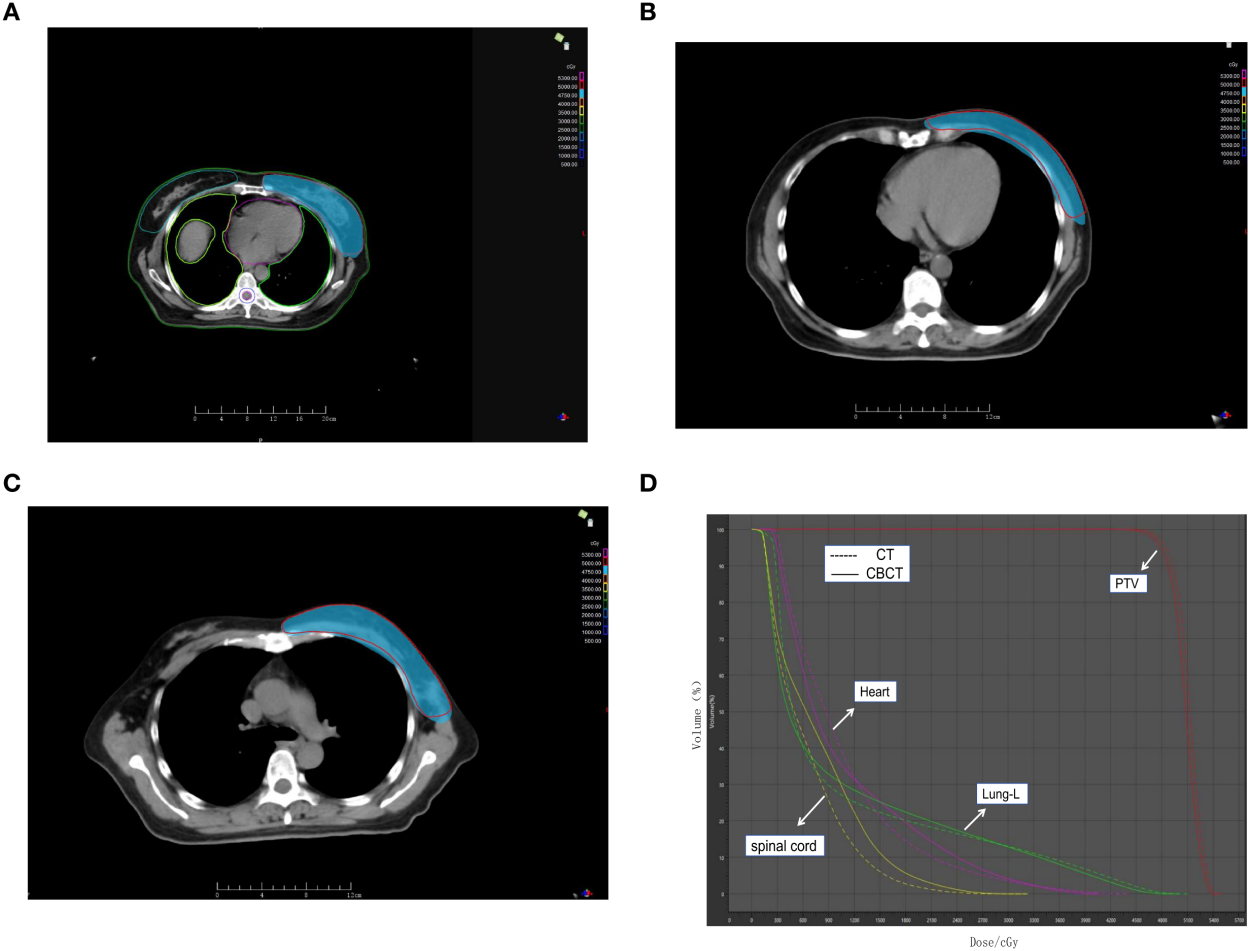
Representative dose distribution and DVH comparison between CT-based and CBCT-based plans. **(A)** Axial dose distribution of CT-based plan showing PTV coverage **(B)** Axial dose distribution of CBCT-based plan showing PTV coverage **(C)**. Axial dose distribution showing delineated PTV and OARs **(D)** Comparison of Dose–Volume Histograms (DVH) for PTV and Organs at Risk (OARs) between CT-based and CBCT-based Plans.

These findings collectively indicate that CBCT-based dose calculations exhibit a high degree of consistency with CT-based planning across both target volumes and critical organs (**Figure 2**). Representative dose distributions and DVH comparisons are presented in **Figure 3**. In addition, representative isodose overlays for two individual patients (Patient 1 and Patient 2) are provided in **Supplementary Figure 3** to further visually support the consistency between CT- and CBCT-based dose calculations.

### Gamma pass rate comparison

Dose verification software was employed to perform two-dimensional gamma analysis for both pCT- and CBCT-based plans, with the detailed results presented in **Table 5**. When the dose threshold was set at 10%, both treatment techniques demonstrated high gamma passing rates. For IMRT, the passing rates under the 3%/3 mm criterion were 97.8% ± 1.6% for the pCT plan and 97.5% ± 1.7% for the CBCT plan; under the more stringent 2%/2 mm criterion, the corresponding values were 93.6% ± 2.5% and 93.1% ± 2.8%. None of the differences were statistically significant (*P* > 0.05). Similarly, for CRT plans, the gamma passing rates under the 3%/3 mm criterion were 98.6% ± 1.2% (pCT) and 98.3% ± 1.3% (CBCT), whereas under the 2%/2 mm criterion, they were 94.5% ± 2.2% and 94.2% ± 2.3%, respectively, with no statistically significant differences observed (*P* > 0.05).

**Table 5:** Statistical results of gamma passing rate for two plans(mean ± SD, %).

Technology	Standard	pCT(%)	CBCT(%)	*t*	*P*
IMRT	3%/3 mm	97.8 ± 1.6	97.5 ± 1.7	−0.598	0.557
	2%/2 mm	93.6 ± 2.5	93.1 ± 2.8	−0.711	0.485
CRT	3%/3 mm	98.6 ± 1.2	98.3 ± 1.3	−0.744	0.467
	2%/2 mm	94.5 ± 2.2	94.2 ± 2.3	−0.538	0.596

### Equivalence analysis

Equivalence between pCT- and CBCT-based dose calculations was assessed using the two one-sided tests (TOST). For all PTV-related dosimetric parameters—including Dmean, D98, D50, D2, HI, and CI—the mean differences and their 90% confidence intervals fell entirely within the predefined equivalence margins (Δ). All TOST *P*-values were below 0.05, demonstrating statistical equivalence between the two calculation methods. The mean differences were small (typically ≤ 0.1 Gy), and none of the 90% CIs approached the equivalence limits. Lin’s CCC and ICC(A,1) exceeded 0.98 across all PTV parameters, indicating excellent agreement. Bland–Altman analyses showed minimal bias with narrow limits of agreement, further supporting the equivalence findings.

Similarly, strong consistency was observed for the OAR parameters. Heart Dmean, V30, V50, as well as Lung Dmean, V20, and V30, all achieved statistical equivalence (TOST *P* < 0.05). The mean differences (0.10–0.30% or 0.10–0.12 Gy) were well below clinically meaningful thresholds. Both CCC and ICC ranged from 0.96 to 0.98, indicating excellent reliability. Bland–Altman plots revealed negligible systematic bias with clinically acceptable limits of agreement (**Table 6**).

**Table 6:** Equivalence analysis between CT- and CBCT-based dose calculations, with dose differences expressed in Gy.

Parameter	Equivalence margin	Mean difference	90% CI of difference	*P-value*	Lin’s CCC	ICC (A,1)	Bland–Altman bias ± LOA
PTV Dmean	± 0.482	0.043	(−0.352,0.438)	0.041	0.993	0.992	0.043 ± 1.274
PTV D98	± 0.491	−0.071	(−0.479,0.329)	0.033	0.991	0.99	0.071 ± 1.346
PTV D50	± 0.468	−0.038	(−0.417,0.341)	0.026	0.995	0.993	0.038 ± 1.106
PTV D2	± 0.493	0.097	(−0.368,0.563)	0.048	0.989	0.987	0.097 ± 1.603
HI	± 0.019	0.001	(−0.007,0.010)	0.013	0.982	0.98	0.001 ± 0.017
CI	± 0.028	−0.011	(−0.025,0.005)	0.046	0.962	0.959	0.011 ± 0.039
Heart Dmean	± 0.537	0.103	(−0.418,0.621)	0.044	0.975	0.973	0.103 ± 1.392
Heart V30 (%)	± 2.186	0.207	(−1.093,1.517)	0.037	0.969	0.965	0.207 ± 2.603
Heart V50 (%)	± 1.087	0.102	(−0.447,0.651)	0.028	0.982	0.981	0.102 ± 1.196
Lung Dmean	± 1.042	0.119	(−0.538,0.777)	0.045	0.979	0.977	0.119 ± 1.547
Lung V20 (%)	± 2.134	0.296	(−1.201,1.796)	0.035	0.972	0.969	0.296 ± 2.784
Lung V30 (%)	± 2.058	0.203	(−1.297,1.704)	0.036	0.974	0.97	0.200 ± 2.695

## Discussion

With the continuous advancement of medical imaging technology, cone-beam computed tomography (CBCT) has become increasingly utilized in radiotherapy. CBCT images can provide real-time three-dimensional anatomical information during treatment, enabling clinicians to better assess the spatial relationship between the target volume and surrounding organs at risk (OARs). Nevertheless, the direct use of CBCT images for dose calculation still faces several challenges, including scatter artifacts, elevated noise levels, limited scan range, and unstable Hounsfield units (HUs), all of which may compromise dosimetric accuracy. To address these issues, various HU correction and calibration strategies have been proposed, and encouraging results have been reported in the literature. These include phantom-based calibration curves (6), density override methods (7, 8)、image registration and histogram matching (9, 10), as well as more recent approaches based on deep learning for synthetic CT (sCT) generation (11, 12).

In this study, we refined the conventional density override method by incorporating patient-specific anatomical information to construct HU-RED calibration curves, which were then applied to CBCT-based dose calculations. Our results demonstrated that differences in planning target volume (PTV) dosimetric parameters (D2, D50, D98, Dmean, homogeneity index [HI], and conformity index [CI]) between CBCT and pCT plans were minimal, with mean deviations below 1.3% and without statistical significance (*P* > 0.05). Similarly, the discrepancies in OAR parameters—including cardiac Dmean, V20, V30, V50, and corresponding pulmonary indices—remained small, with mean differences under 1.5%. The largest variation was observed in cardiac V20 in one patient (2.1%). Two-dimensional gamma analysis under both 3%/3 mm and 2%/2 mm criteria further confirmed the high consistency of dose distributions between the two plans. These findings are in agreement with previous reports by (13)、Hamming (14) as well as Maspero (15), which collectively support the clinical feasibility of using corrected CBCT images for treatment dose calculation. When comparing different irradiation techniques, namely IMRT and CRT, no statistically significant differences were identified between CBCT- and pCT-based plans. This suggests that HU-RED–corrected CBCT images can be applied reliably across different radiotherapy modalities. Consistent with earlier studies, our results indicate that the accuracy of CBCT dose calculations is primarily influenced by HU calibration methods and registration strategies, rather than the specific delivery technique employed (16, 17).

In terms of OAR dosimetry, the overall differences in cardiac and pulmonary doses were modest. Compared with other tumor sites, breast radiotherapy is more susceptible to respiratory motion and setup variations, which may explain localized deviations in some cases. Prior investigations have shown that respiratory motion can alter chest wall and cardiac positions, thereby affecting dose distribution (18, 19). In our study, however, the use of rigid registration and selection of pre-treatment CBCT scans minimized anatomical discrepancies, ensuring that dose variations remained within clinically acceptable limits.

It is noteworthy that recent advances in artificial intelligence and deep learning have brought CBCT quality enhancement and sCT generation into the spotlight. Several studies have confirmed that deep learning-based sCT reconstruction can effectively reduce artifacts, improve HU consistency, and yield CBCT-based dose calculations that closely approximate those of CT (20–22). This emerging direction offers promising potential for adaptive radiotherapy in breast cancer.

In addition to the overall dosimetric consistency observed between CBCT- and CT-based plans, thisstudy has several notable strengths. First, we applied a patient-specific HU-RED calibrationapproach that integrates individual anatomical characteristics, which is more accurate thanconventional phantom-based or global density override methods. Second, the dosimetric comparison was not limited to traditional paired testing; instead, we implemented a comprehensive agreement assessment that included TOST equivalence testing, Lin’s CCC, ICC(A,1), and Bland–Altman analysis. The consistent finding of statistical equivalence (TOST *P* < 0.05) and the uniformly high concordance coefficients further strengthen the robustness and reliability of our results. These methodological enhancements provide a higher level of evidence regarding the feasibility of HU-RED–corrected CBCT for clinical dose calculation. Despite these advantages, the present study has certain limitations. First, the sample size was relatively small, which may limit the generalizability of the findings and warrants confirmation in larger, multicenter studies. Second, only rigid registration was performed, without accounting for anatomical deformations that may arise from respiratory motion or setup variability; such changes could influence dose accuracy in daily treatment scenarios. Although rigid registration may not fully capture local deformation, the supplementary ROI-based HU and RED comparisons (**Supplementary Figures 1**, **S2**) suggest that, at the tissue-averaged ROI level, the residual HU/RED discrepancies between pCT and CBCT remain limited, which is consistent with the observed dosimetric agreement in this cohort. Third, our investigation focused exclusively on breast cancer, and whether this HU-RED calibration strategy can be directly applied to other anatomical sites (e.g., head and neck, pelvis) remains to be established. Future work should incorporate deformable registration and advanced deep-learning–based sCT generation to develop more universal HU-RED calibration frameworks.

In summary, our findings demonstrate that HU-RED–corrected CBCT images yield dose calculation results highly consistent with pCT in breast radiotherapy, supporting their use for clinical dosimetric evaluation. With continued improvements in CBCT imaging and the integration of deep learning technologies, more accurate adaptive radiotherapy may become feasible in the near future.

## Data Availability

The datasets generated and analyzed during the current study are available from the corresponding author on reasonable request.

## Supplementary material

Supplementary Figure 1 ROI-based mean HU comparison.

Supplementary Figure 2 ROI-based mean RED comparison.

Supplementary Figure 3 Representative isodose distribution overlays for two patients. **(A)** CBCT-based dose distribution for Patient 1. **(B)** CT-based dose distribution for Patient 1. **(C)** CBCT-based dose distribution for Patient 2. **(D)** CT-based dose distribution for Patient 2. Isodose lines are shown to illustrate the agreement between CT- and CBCT-based dose calculations.
