# Supplementary figures and images for: Evaluation of the accuracy of cone-beam CT–based dose calculation for target volumes and organs at risk in left-sided breast cancer radiotherapy
Source: Front Oncol. 2026 Mar 3;16:1768319. doi: 10.3389/fonc.2026.1768319 (PMC12996974; doi:10.3389/fonc.2026.1768319)

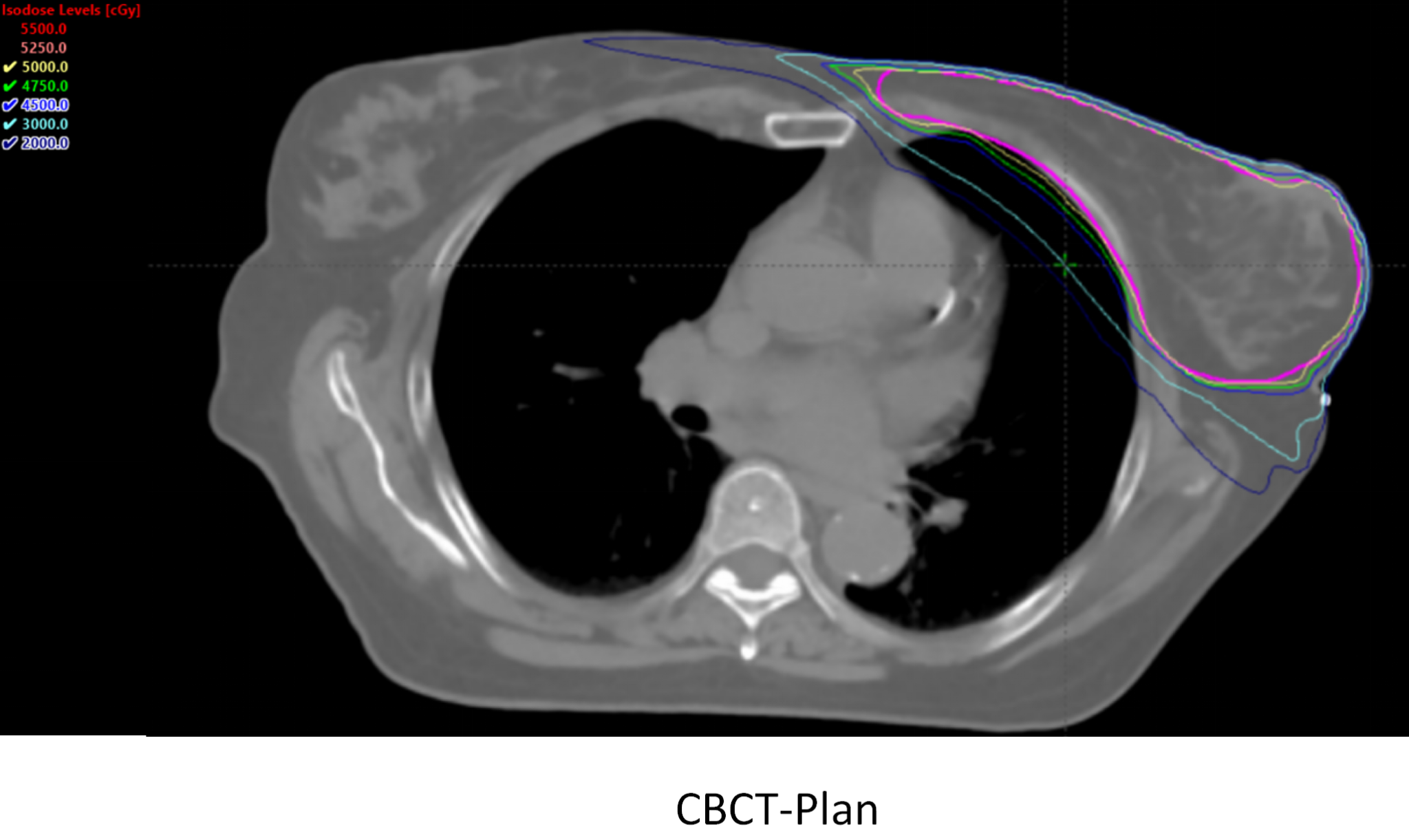

Supplement: Supplementary Figure 1 — ROI-based mean HU comparison. [file Image1.tiff]

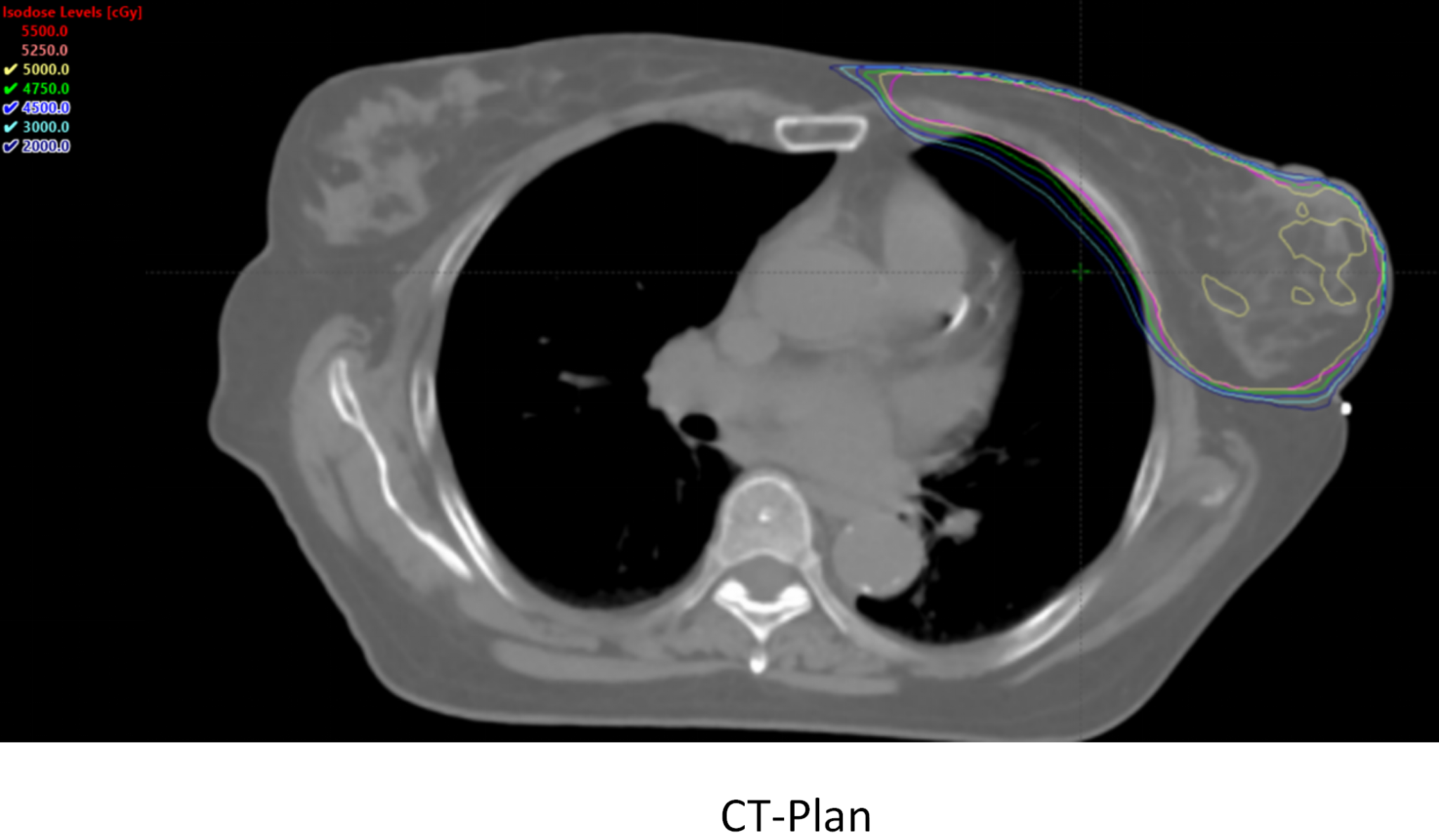

Supplement: Supplementary Figure 2 — ROI-based mean RED comparison. [file Image2.tiff]

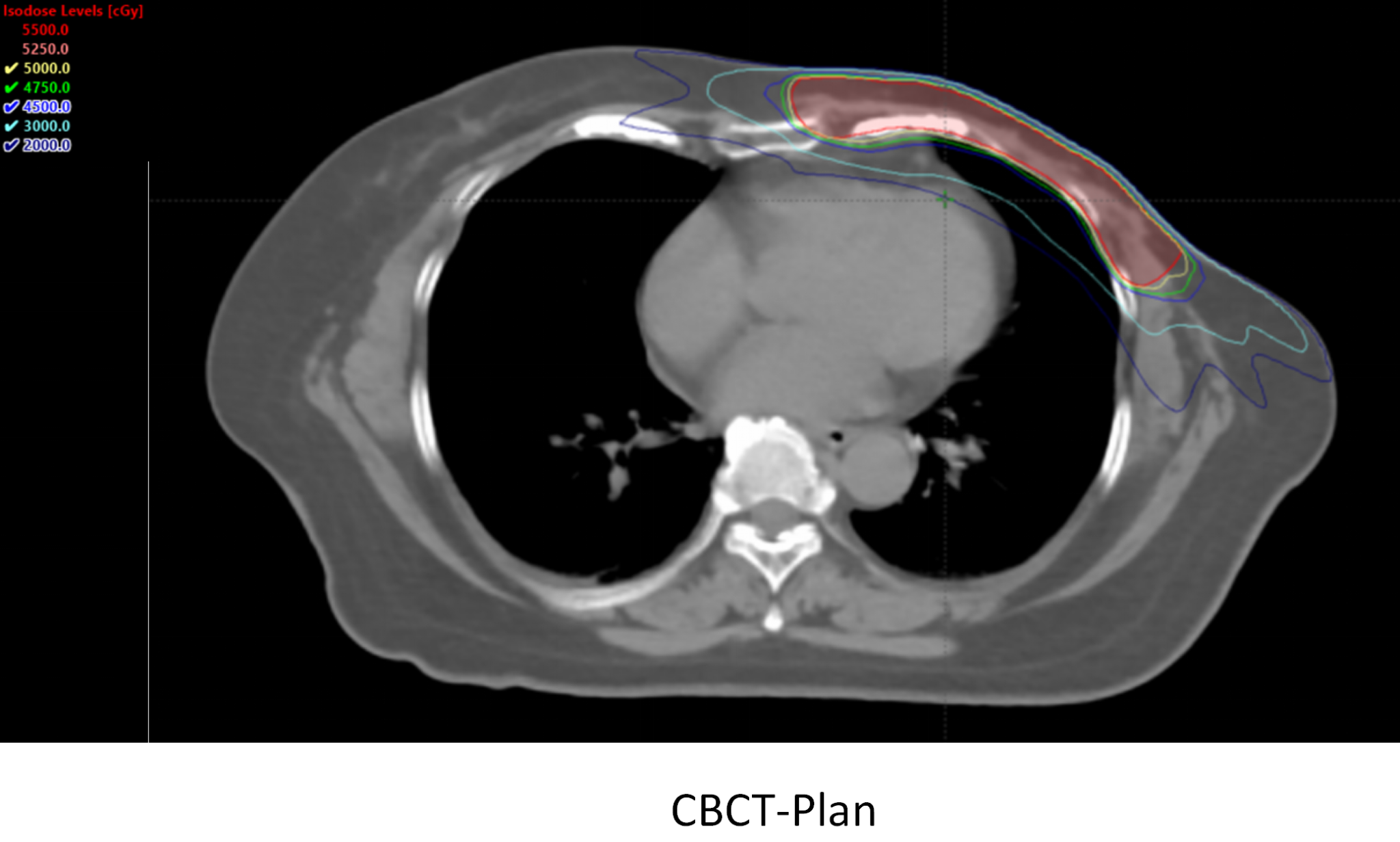

Supplement: Supplementary Figure 3 — Representative isodose distribution overlays for two patients. (A) CBCT-based dose distribution for Patient 1. (B) CT-based dose distribution for Patient 1. (C) CBCT-based dose distribution for Patient 2. (D) CT-based dose distribution for Patient 2. Isodose lines are shown to illustrate the agreement between CT- and CBCT-based dose calculations. [file Image3.tiff]

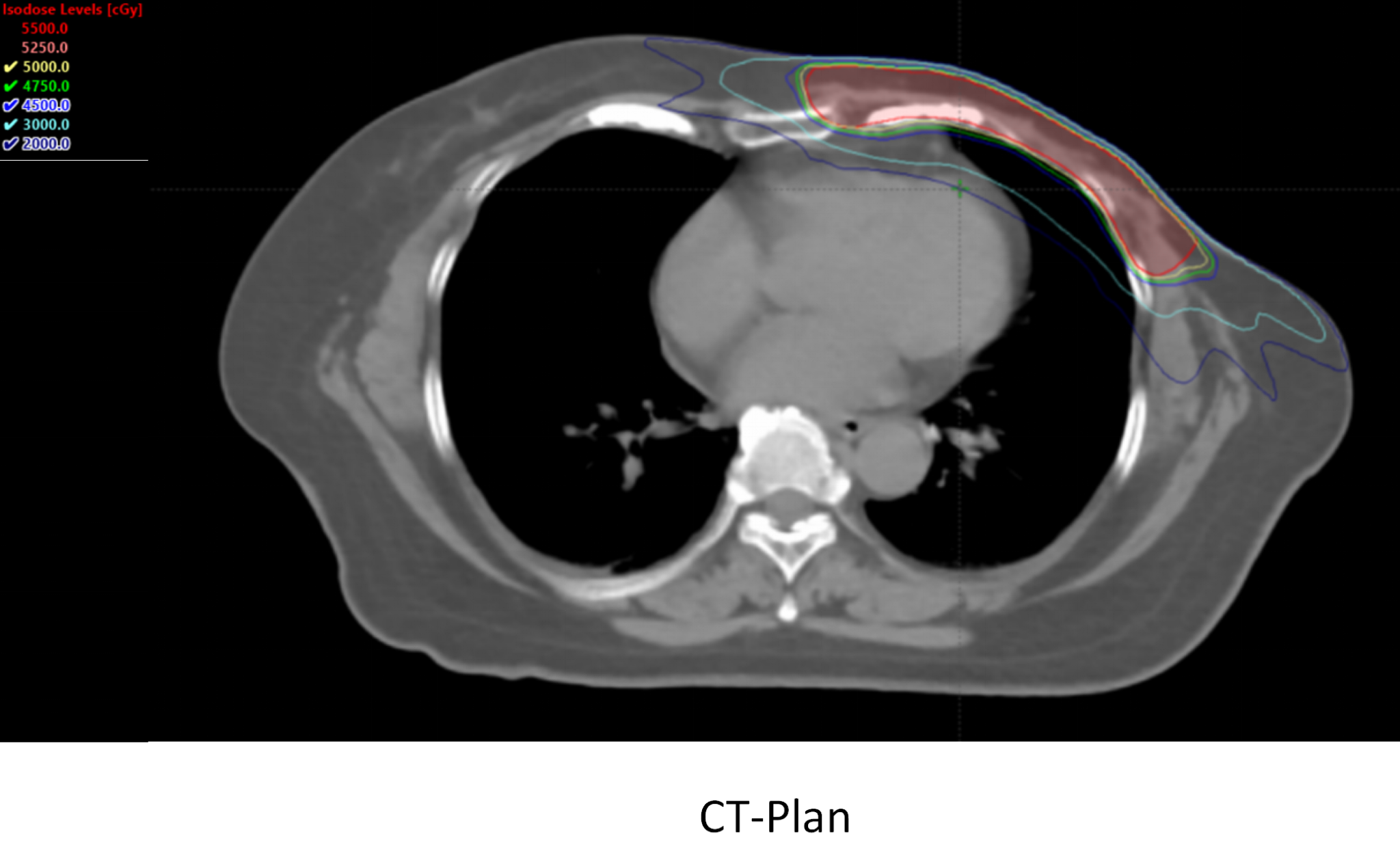

Supplement: Supplementary file 4 [file Image4.tiff]

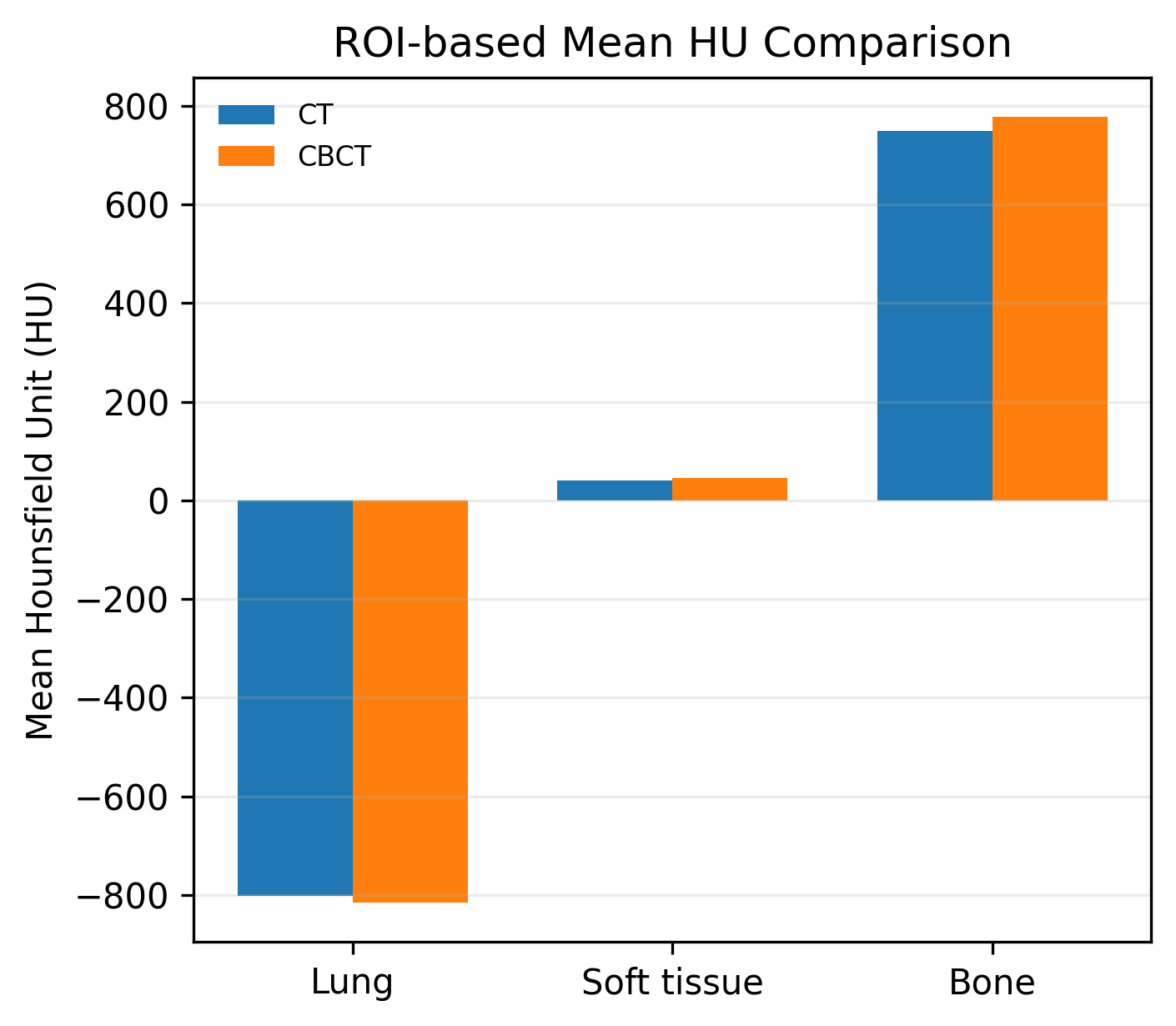

Supplement: Supplementary file 5 [file Image5.tiff]

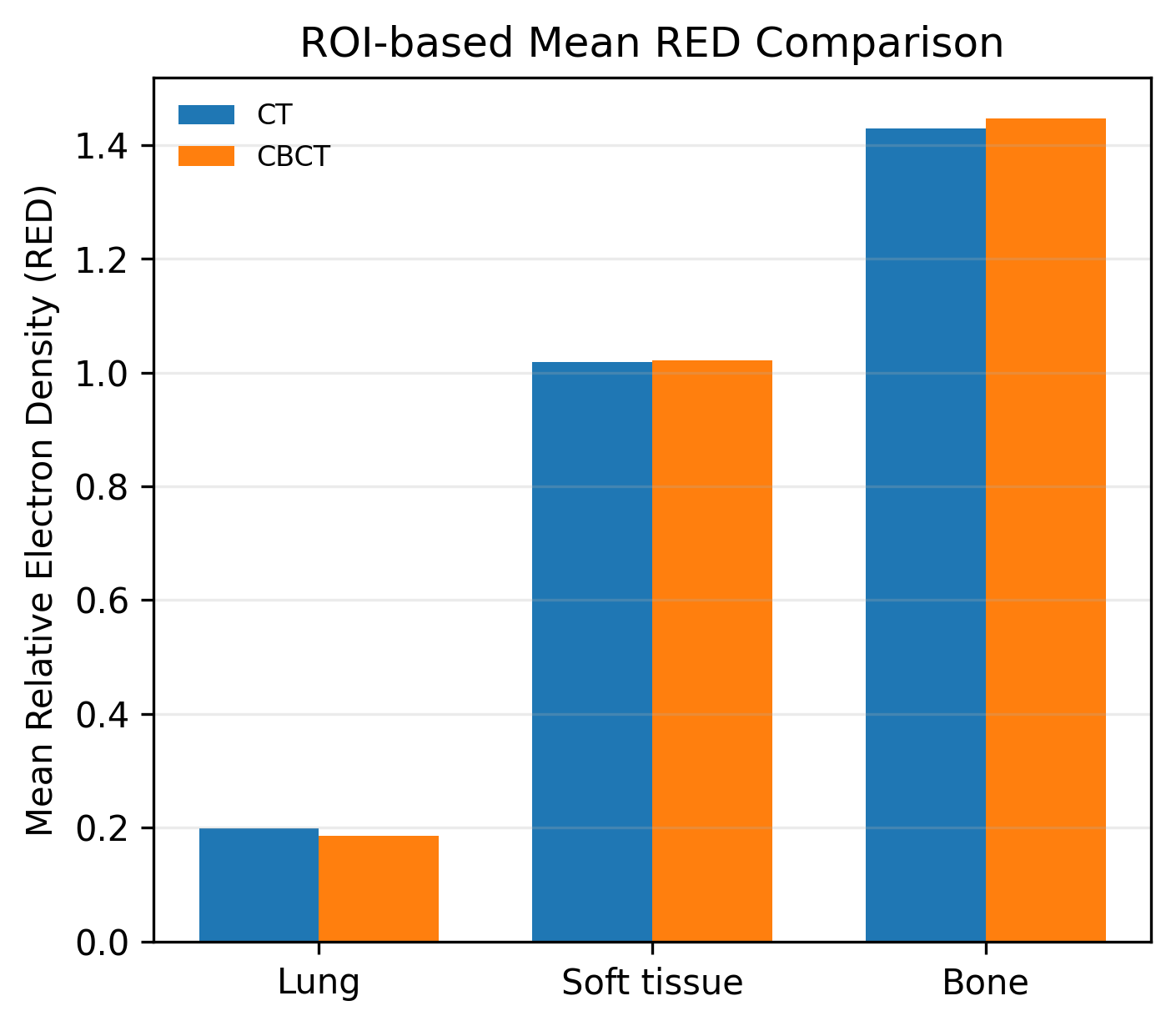

Supplement: Supplementary file 6 [file Image6.tiff]
